# Sensitization to timothy grass pollen allergenic molecules in children

**DOI:** 10.1186/2049-6958-8-17

**Published:** 2013-03-01

**Authors:** Alessandra Scaparrotta, Marcello Verini, Nicola Pietro Consilvio, Anna Cingolani, Daniele Rapino, Marina Attanasi, Marzia Cerasa, Sabrina Di Pillo, Francesco Chiarelli

**Affiliations:** 1 Department of Pediatrics, University of Chieti, Via Dei Vestini 5, 66100, Chieti, Italy

**Keywords:** Children, Grass pollen allergy, Phleum pratense, Recombinant allergen, rPhl p 1, rPhl p 5

## Abstract

**Background:**

Grass pollens are significant elicitors of IgE-mediated allergic disease in the world and timothy (Phleum pratense) is one of the most important pollens of the family. Molecular and biochemical characterization of Phleum pratense has revealed several allergen components: rPhl p 1 and rPhl p 5 have been shown to be “Species Specific Allergens”, while the profilin rPhl p 12 and the calcium-binding protein rPhl p 7 are the principal Cross-Reactive components.

**Methods:**

In this study the pattern of sensitization to rPhl p 1, rPhl p 5, rPhl p 7 and rPhl p 12 was analyzed in children with asthma and/or rhinoconjunctivitis and grass pollen allergy, in order to evaluate the frequency of sensitization to allergenic molecules of Phleum pratense among pediatric subjects allergic to grass pollen in a Mediterranean population. The correlation of sensitization to these Phleum allergenic molecules with IgE against grass pollen extract and its variation according to age and level of IgE against grass pollen extract were evaluated.

**Results:**

IgE against to rPhl p 1 were found in 99% (205/207) of patients, to rPhl p 5 in 67% (139/207), to rPhl p 12 in 32% (66/207) and to rPhl p 7 only in 5% (10/207).

Sensitization only to “Species Specific” (rPhl p1, rPhl p5) allergenic molecules of Phleum pratense was detected in 65% (135/207) of children. Our data show the predominant role of rPhl p 1 in pediatric populations as the most relevant sensitizing allergen detectable at all ages and at all levels of timothy grass pollen-specific IgE antibodies, while the importance of rPhl p 5 rises with the increase of patients’ age and with grass pollen IgE levels.

**Conclusions:**

The assessment of sensitization to grass pollen allergenic molecules could help develop a better characterization of allergic sensitization in grass pollen allergy in children, which may be different in every patient. It could also enable clinicians to give more specific and effective immunotherapy, based on allergenic molecule sensitization.

## Background

Traditional allergen extracts, used for diagnosis and therapy, are prepared from natural allergen sources such as a mixture of different grass species, and contain mixed allergenic components in undefined amounts of non allergenic materials. These components are difficult to standardize and in many cases important allergens are present in small amounts or lacking, such as their biological potency is subject to wide variability [1,2]. On the contrary, recombinant allergens can be produced with high purity by using controlled procedures that yield defined molecules with known molecular, immunologic, and biological characteristics [3,4].

In the late 1980s the rapid development of molecular biological techniques allowed the cloning of the first molecular allergen [5], and the subsequent advent of recombinant technology provided a large panel of allergenic molecules [1,6-8].

Grass pollens are important causes of IgE-mediated allergic disease in the world and approximately 40% of allergic patients show IgE reactivity to these allergens [9-11]. Timothy grass (Phleum pratense or Pp) is the most important source of grass pollen allergens in northern and central Europe [12,13].

Molecular and biochemical characterization of Pp [12] has revealed several allergen components as rPhl p 1, rPhl p 2, nPhl p 4, rPhl p 5, rPhl p 6, rPhl p 7, rPhl p 11 and rPhl p 12, of which rPhl p 1 and rPhl p 5 have been shown to be the “Species Specific Allergens” [14,15]; the profilin rPhl p 12 [12,16] and the calcium-binding protein rPhl p 7 are the main Cross-Reactive components.

The major part of the studies conducted to assess the utility of molecular diagnosis based on recombinant allergens have been performed on a sample of population with a very wide range of ages (from childhood to adulthood); few epidemiological studies have been performed exclusively in the pediatric age.

In this study, the pattern of sensitization to rPhl p 1, rPhl p 5, rPhl p 7 and rPhl p 12 in children with asthma and/or rhinoconjunctivitis and grass pollen allergy (positive IgE against timothy grass pollen extract) was analyzed, in order to evaluate the frequency of sensitization to allergenic molecules of Pp in children with pollen allergy in a Mediterranean population.

The correlation of sensitization to these Pp allergenic molecules with IgE against grass pollen extract and its variation according to different ages and different levels of IgE against grass pollen extract were evaluated.

## Methods

### Study population

207 patients (62 females and 145 males), mean age 9.7 ± 3.6 years, referring to the Allergy and Respiratory Unit of Pediatric Clinic, University Hospital, Chieti, Italy, were recruited between August 2008 and April 2010.

The selection was based on a positive history of allergic asthma and/or rhinoconjunctivitis and timothy grass pollen (Pp) allergy, confirmed by allergological evaluation (skin prick tests and Pp specific IgE positivity).

All patients were polysensitized to other respiratory and food allergens, listed in Table 1.

**Table 1:** General, allergological and clinical characteristics of the study population

***Characteristics of population***	**N° of patients (%)**
Number of patients	207
Males	145
Females	62
Mean Age (years)	9.7 ± 3.6
**Respiratory Allergy**	
Timothy Grass Pollen	207/207
Parietaria judaica	150/207 (72%)
Olea	176/207 (85%)
House dust mite	108/207 (52%)
Moulds	58/207(28%)
Dog/cat epithelia	85/207 (41%)
**Food Allergy**	
Peanuts	77/207 (37%)
Tomato	104/207 (50%)
Milk	13/207 (6%)
Eggs	19/207 (9%)
**Allergic Symptoms**	
Rhinitis	147/207 (71%)
Asthma	125/207 (60%)
Conjunctivitis	43/207 (21%)
Urticaria, Angioedema, Anaphylaxis	24/207 (12%)

The ethical committee of the University of Chieti approved the study performed in accordance with Helsinki Declaration (1964); written informed consent was obtained from the parents of all patients.

### Methods

Serum specific IgE against timothy grass pollen (Pp) extract and serum specific IgE against Pp allergenic molecules (rPhl p 1, expansin family; rPhl p 5, ribonuclease family; rPhl p 7 polcalcin family; and rPhl p 12 profilin family) were measured using ImmunoCAP (Phadia AB, Uppsala, Sweden).

IgE against grass pollen extract and IgE against allergenic molecules of Pp were scored according to the RAST rating: RAST rating 1 (0.35 - 0.69 kUA/l); RAST rating 2 (0.70 - 3.49 kUA/l); RAST rating 3 (3.50 - 17.49 kUA/l); RAST rating 4 (17.50 - 49.99 kUA/l); RAST rating 5 (50.0 - 100.00 kUA/l); RAST rating 6 (> 100 kUA/l). IgE were considered positive at the level of 0.35 kUA/l (class I or RAST rating 1) or higher.

Total IgE were considered high according to the reference age-related value of ImmunoCAP Total IgE, Phadia.

The correlation between IgE against Pp allergenic molecules and IgE against timothy grass pollen (Pp) extract was analyzed.

The frequency of patients with positive serum IgE against Pp allergenic molecules was correlated with serum IgE against grass pollen (Pp) extract (according to RAST scores), in order to verify an IgE cut-off of sensitization to rPhl p 1 and rPhl p 5 IgE.

The frequency of sensitization to Pp allergenic molecules according to the age was also studied, dividing the patients in three groups: age < 5 years; age between 5 to 10 years; age > 10 years.

We also analyzed the sensitization to other allergens in patients with positivity to “Species Specific” allergenic molecules (rPhl p 1, rPhl p 5) and Cross-Reactive allergenic molecules, comparing the pattern of polysensitization with those of patients sensitized only for “Species Specific” allergenic molecules.

Finally, possible correlations between clinical symptoms (asthma, rhinitis, rhinoconjunctivitis, urticaria, anaphylaxis, angioedema) and sensitization to Pp allergenic molecules were explored.

### Statistical analysis

Results are expressed as Mean (M) ± Standard Deviation (SD). Mean values are compared using unpaired *T* Test. P values < 0.05 are considered statistically significant. The Pearson correlation coefficient was used to find correlations between allergological and laboratory parameters. Differences between groups of patients have been analyzed using the Chi square (*χ*^2^) Test and Yates’ corrected Chi square (Yates’ *χ*^2^) Test. SPSS Inc PASW Statistics 18 was used for the statistical analysis.

## Results

High total IgE levels in relation to the age were found in 91% (189/207) of patients.

The frequency of allergic symptoms in our patients is listed in Table 1.

**Figure 1:**
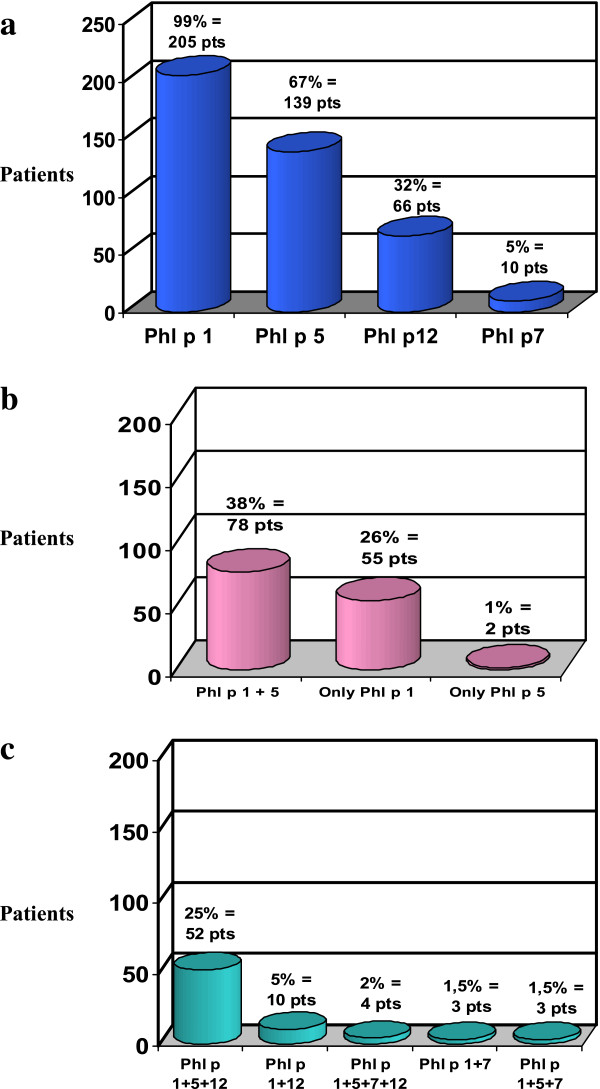
**Pattern of sensitization to allergenic molecules of Pp in 207 timothy grass pollen allergic children.** Number and percentage of patients with: **a**) sensitization to all allergenic molecules; **b**) sensitization to “Species Specific” allergenic molecules alone; **c**) sensitization to “Species specific” and Cross-Reactive allergenic molecules.

IgE against rPhl p 1 were found in 99% (205/207) of patients, against rPhl p 5 in 67% (139/207), against rPhl p 12 in 32% (66/207) and against rPhl p 7 only in 5% (10/207) (Figure 1a).

Sensitization only to “Species Specific” allergenic molecules of Pp was detected in 65% (135/207) of children: in particular, sensitization only to rPhl p 1 was found in 55/207 (26%) patients, only to rPhl p 5 in 2/207 (1%) children and to rPhl p 1 + rPhl p 5 in 78/207 (38%) patients (Figure 1b).

No children had positive IgE only against rPhl p 7 and rPhl p 12.

Sensitization to “Species Specific” and Cross-Reactive allergenic molecules of Pp was detected in 35% (72/207) of patients: IgE against rPhl p 1 + rPhl p 5 + rPhl p 12 were found in 25% of patients (52/207); against rPhl p 1 + rPhl p 12 in 5% (10/207 ones); against rPhl p 1 + rPhl p 5 + rPhl p 7, and against rPhl p 1 + rPhl p 7 in 1.5% (3/207 patients. Simultaneous positivity to all four allergenic molecules was detected in a very small number of children (rPhl p 1 + rPhl p 5 + rPhl p 7 + rPhl p 12 in 4/207**;** 2%) (Figure 1c).

High significant correlations were observed between IgE levels against timothy grass pollen extract and: rPhl p 1 IgE (Pearson coefficient: 0.87, p = 0.01); rPhl p 5 IgE (Pearson coefficient: 0.77, p = 0.01)**,** whereas lower correlations were found with rPhl p 7 IgE (Pearson coefficient: 0.19, p = 0.01) and rPhl p 12 IgE (Pearson coefficient: 0.36, p = 0.01).

When we analyzed IgE levels against timothy grass pollen extract and RAST rating we found that 24/207, 37/207, 41/207, 43/207 and 62/207 patients had a RAST rating of 2, 3, 4, 5 and 6, respectively.

**Figure 2:**
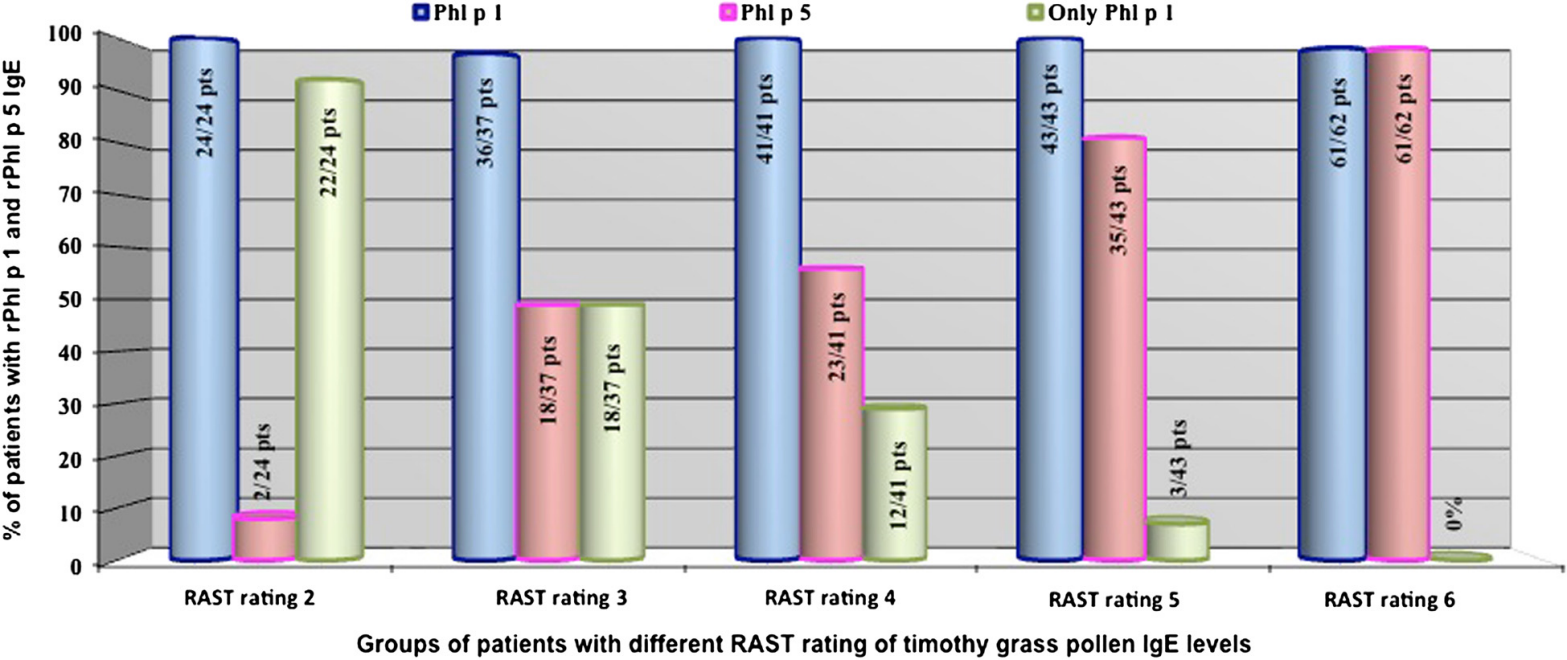
**IgE positivity for Phleum “Species Specific” allergenic molecule according to grass pollen IgE levels in timothy grass pollen allergic children.** Abbreviation: pts = patients.

rPhl p 1 IgE were detectable from 97% to 100% as in patients with low grade of sensitization as in high sensitized ones, regardless of the level of IgE against timothy grass pollen extract (Figure 2). So, there were no statistically significant differences in the positivity of IgE for rPhl p 1 according to IgE against timothy grass pollen extract levels.

rPhl p 5 IgE positivity raised with the increase of IgE against timothy grass pollen extract (Figure 2), with statistically significant differences (Table 2).

**Table 2:** Comparison of the positivity for Phleum allergenic molecules IgE between groups of patients with different IgE values against timothy grass pollen extract levels

	**rPhl p 5 IgE**			**Only rPhl p 1 IgE**			**rPhl p 12 IgE**		
**GRASS POLLEN IgE GROUPS**	***χ***^**2**^	**Yates’ *χ***^**2**^	**p**	***χ***^**2**^	**Yates’ *χ***^**2**^	**p**	***χ***^**2**^	**Yates’ *χ***^**2**^	**p**
**RAST rating 2 vs 3**	10.7	9	p < 0.01	11.9	10.1	p < 0.01	--	--	ns
**RAST rating 2 vs 4**	14.6	12.6	p < 0.01	23.6	21.2	p < 0.01	8.6	6.8	p < 0.01
**RAST rating 2 vs 5**	33.2	30.4	p < 0.01	47.2	43.7	p < 0.01	11.7	9.8	p < 0.01
**RAST rating 2 vs 6**	71.6	67.1	p < 0.01	76.4	71.6	p < 0.01	25.1	22.8	p < 0.01
**RAST rating 3 vs 4**	--	--	ns	--	--	ns	9.9	8.1	p < 0.01
**RAST rating 3 vs 5**	9.5	8.1	p < 0.01	17.8	15.7	p < 0.01	14.1	12.2	p < 0.01
**RAST rating 3 vs 6**	35.6	32.5	p < 0.01	36.9	33.7	p < 0.01	31.8	29.4	p < 0.01
**RAST rating 4 vs 5**	6.3	5.2	p < 0.05	7.11	5.7	p < 0.01	--	--	ns
						p < 0.05			
**RAST rating 4 vs 6**	29.3	26.6	p < 0.01	20.5	17.8	p < 0.01	9.1	8	p < 0.01
**RAST rating 5 vs 6**	9.3	7.3	p < 0.01	4.4		p < 0.05	5.1	4.3	p < 0.05

Monosensitization to rPhl p 1 was very important in patients with lower IgE against timothy grass pollen extract levels and it disappeared with the increase of IgE against timothy grass pollen extract level (Figure 2), with statistically significant differences (Table 2).

rPhl p 5 IgE positivity became the same as rPhl p 1 in patients with IgE against timothy grass pollen extract level ≥ 100 kUA/l (Figure 2).

rPhl p 12 IgE positivity raised with the increase in IgE against timothy grass pollen extract, with statistically significant differences (Table 2).

Figure 2 shows IgE positivity for rPhl p 1, rPhl p5 and only rPhl 1 according to timothy grass pollen IgE levels (Figure 2), while Table 2 summarizes the statistical significance that emerges comparing the positivity for Pp allergenic molecules IgE between groups of patients with different IgE against timothy grass pollen extract levels (Table 2).

The patients aged < 5 years were 17/207 (8%), 12 males and 5 females, those aged between 5 to 10 years were 102/207 (49%), 68 males and 34 females, and those aged > 10 years 88/207 (43%), 65 males and 23 females. Hereinafter we report the pattern of specific IgE to rPhl p 1, rPhl p 5, rPhl p 7 and rPhl p 12 in children divided in three groups according to the age **(**the first group < 5 years, the second group between 5 and 10 years, and the third group > 10 years): rPhl p 1 in 100% (17/17), 99% (101/102) and 99% (87/88) in the 1^st^, 2^nd^, and 3^rd^ group, respectively; rPhl p 5 in 59% (10/17), 67% (68/102) and 69% (61/88), respectively; rPhl p 7 in 0%, 6% (6/102) and 4% (4/88); rPhl p 12 in 30% (5/17), 28% (29/102) and 36% (32/88); and finally mononosensitization only to rPhl p 1 was detected in 35% (6/17), 28% (29/102) and 23% (20/88)**,** while only to rPhl p 5 in 0%, 1% (1/102) and 1.1% (1/88), respectively.

Sensitization to rPhl p 1 was detected in almost all patients at all ages, and there was no significant increase in the frequency of positivity to rPhl p 5 and to rPhl p 12 with the rise of the age. Monosensitization to rPhl p 1 decreased with the increase in the children’s age, although without statistical significance (with Chi square test).

Dividing the patients in two groups according to sensitization to “Species Specific” allergenic molecules only (rPhl p 1, rPhl p 5; SS group with 135/207 patients) and to “Species Specific” and Cross-Reactive allergenic molecules (rPhl p 1, rPhl p 5, rPhl p 7 and rPhl p 12; SSCR group with 72/207 patients), we evaluated the most relevant pattern of respiratory and food sensitization of our population of polysensitized children.

rPhl p 1 was found in 133/135 (98.5%) of SS group patients and in 72/72 (100%) of the SSCR group, while rPhl p 5 was found in 59% (80/135) of group SS patients vs 82% (59/72) of the other group, with a significant difference (*χ*^2^ =10.95; Yates’ *χ*^2^ = 9.95; p<0.01).

Children with IgE against “Species Specific” and Cross-Reactive allergens were more frequently sensitized to Parietaria judaica and Olea as aeroallergens and to peanuts and tomato as foods in comparison to patients with IgE against “Species Specific” allergenic molecules only, with statistical significance (Table 3).

**Table 3:** Pattern of sensitization against to other aeroallergens (Parietaria and Olea) and foods (peanuts and tomato), according to the positivity of IgE for only “Species Specific” allergenic molecules (SS group) and “Species Specific” and Cross-Reactive (SSCR group) allergens of Pp

**IgE Positivity against:**	**“SPECIES SPECIFIC” ALLERGENIC MOLECULES**	**“SPECIES SPECIFIC” AND CROSS-REACTIVE ALLERGENIC MOLECULES**	***χ***^**2**^	**Yates’ *χ***^**2**^	**P**
**Parietaria Judaica**	82/135 patients (61%)	68/72 patients (94%)	26.7	25.1	< 0.01
**Olea**	105/135 patients (78%)	71/72 patients (99%)	16.1	14.4	< 0.01
**Peanuts**	30/135 patients (22%)	47/72 patients (65%)	8.9	8.1	< 0.01
**Tomato**	46/135 patients (34%)	58/72 patients (80%)	40.6	38.7	< 0.01

Par j 2 IgE levels were not evaluated in all patients sensitized to Parietaria, but it was particularly interesting to note that in patients without IgE against Cross-Reactive allergenic molecules (SS group), the majority of patients (with positive IgE for Parietaria) in whom Par j 2 IgE levels were studied (36/41 patients = 88%) were highly sensitized to Parietaria; while only 50% of patients (18/36) with IgE against Parietaria had also IgE against Par j 2 in SSCR group (*χ*^2^=11.2, Yates’ *χ*^2^ = 9.6, p > 0.01).

It is also very remarkable that patients with IgE only against “Species Specific” allergenic molecules of Pp did not have IgE vs Bet v 2 (another profilin, tested on 44 patients of SS group), while IgE against Bet v 2 were found in 83% (25 patients on 30, in which the profilin was tested) of SSCR group, with statistical significance (*χ*^2^ = 55.4, Yates’ *χ*^2^ = 51.7, p < 0.01).

There weren’t significant differences in clinical symptoms such as asthma, rhinitis and conjunctivitis according to different sensitization to Pp allergenic molecules (SS group vs SSCR group). On the other hand, patients who also had Cross-Reactive allergens (SSCR group) were more often affected by anaphylaxis, urticaria and angioedema caused by peanuts, tomato and fruits (14/72 = 19.4% on SSCR vs 10/135 = 7% of SS group), with statistical significance (*χ*^2^ = 6.64, p < 0.01; Yates’ *χ*^2^ = 5.52, p < 0.05).

## Discussion

Recombinant allergens can be produced as molecules that exactly mimic the properties of the natural allergens**,** or modified variants with advantageous properties such as reduced allergenic activity or increased immunogenicity, or alternatively as hybrid molecules resembling the epitopes of several different allergens to include the relevant epitopes of complex allergen sources [3]. Recombinant allergens can be used instead of timothy grass pollen extracts, that exhibit a considerable heterogeneity regarding the presence of individual allergens and hence yield a certain variability of the results of the *in vivo* test. A single recombinant allergen or a combination of a few major recombinant allergens can substitute the crude extract for diagnostic purposes *in vitro* and *in vivo*[17].

rPhl p 1 is considered by many authors the most important timothy grass pollen “Species Specific” allergen [18-22]**,** whereas other authors maintain that the rPhl p 5 is the most significant [23-27].

In a recent study, Casquete - Román *et al*. detected in a pediatric population a total of 99.4% of the patients classified as sensitized to grass pollen who yielded positive values (> 0.1 kUA/l) for the recombinant “Species Specific” allergens (rPhl p 1 + rPhl p 5), while 46% of them proved positive for the Cross-Reactive (rPhl p 7 + rPhl p 12) allergens [1]. Rossi *et al.* found the following frequency of sensitization in 77 patients (mean age 21.6 years): rPhl p1 = 93.5%; rPhl p 5 = 72.7%; rPhl p 7 = 7.8%; rPhl p 12 = 35.1% [14]. In a subsequent very recent study (2010), Rossi detected in 33 patients (age range 9–62 years) rPhl p 1 in 100% of patients, rPhl p 5 in 76%, rPhl p 7 in 3% and rPhl p 12 in 45% [28]. Mari found the following data in sera of 749 grass-sensitized patients, selected on a population of 4,606 unselected subjects, with an age range of 2 to 70 years: rPhl p 1 = 83%, rPhl p 5 = 50%, rPhl p 7 = 7%, rPhl p 12 = 15% and isolated reactivity to rPhl p 1 in 6%, whereas it was negligible for the remaining molecules [15].

Among the most important recombinant allergens we measured rPhl p 1 and rPhl p 5, because they are considered the major “Species Specific Allergens” in the international literature [29-31] and rPhl p 12 and rPhl p 7, the principal Cross-Reactive components. Our study has shown the predominance in children with grass pollen allergy of both of rPhl p 1 and rPhl p 5, found in 99% and 67% of them, respectively.

The pan-allergen rPhl p 12 was detected in a notable percentage of children (32%), while Valenta et al. [32] reported that 20% of patients allergic to grass displayed IgE reactivity to profilins.

The discrepancy between these frequencies of sensitization is probably related to a geographical variation in allergen exposure.

Sensitization only to “Species Specific” allergenic molecules of Pp was observed very frequently, in more than half of children (65%), while sensitization to “Species Specific” and Cross-Reactive allergenic molecules together was found in a smaller percentage (35%). This may reflect the impact of large amounts of grass pollen in this geographic area, as observed also by Rossi et al. [33].

rPhl p 1 was the only allergenic molecule of Pp detected alone in a relevant percentage of patients (26%), while rPhl p 5 was rarely found as the only sensitizing allergen (1%). No patients were uniquely sensitized against rPhl p 7 and rPhl p 12. Consequently, this study has shown that rPhl p 1 is the predominant grass pollen allergenic molecule, sensitizing almost all patients both in association with other rPhl and acting as a single allergen.

There was a significant correlation between serum IgE against timothy grass pollen extract and Pp IgE levels.

Laffer *et al*. examined in sera of 183 patients allergic to grass pollen from different populations (Europe, Japan, and Canada) the *in vitro* IgE antibody-binding capacity to three recombinant timothy allergens, rPhl p 1, rPhl p 2, rPhl p 5, and birch profilin. More than 94% of patients could be diagnosed with a combination of recombinant rPhl p 1, rPhl p 2, rPhl p 5, and birch profilin, while the sera that did not react with the recombinant allergens contained low levels of timothy grass pollen-specific IgE antibodies. A good correlation was observed between natural timothy-serum specific IgE antibodies and the combination of recombinant allergens in all 183 tested sera [34].

The analysis of the IgE positivity for Pp allergenic molecules according to timothy grass pollen-specific IgE levels showed that rPhl p 1 IgE were detectable at any level of grass pollen IgE (in 97% - 100% of patients), both in children with a low grade of sensitization and in high sensitized grass pollen patients, while monosensitization to rPhl p 1 was very important in patients with lower grass pollen IgE levels, disappearing with the increase in timothy grass pollen-specific IgE antibodies (with statistical significance). However, rPhl p 5 IgE positivity was raised with the increase in grass pollen IgE level, with statistically significant differences, and it became the same as rPhl p 1 in patients with higher grass pollen IgE level (≥ 100 kUA/l). Moreover, rPhl p 12 IgE positivity increased with IgE against timothy grass pollen extract, once again with significant differences.

Studying the pattern of sensitization to Pp allergens according to the patients’ age, we have observed some interesting age-related differences. Sensitization to rPhl 1 seems to appear early, even before the age of 5 years, because it was found in 100% of our patients at this age. rPhl p 5 sensitization, although detected in a smaller percentage of children at the age < 5 years, was already present at this age and its positivity increased with the rise of the age (from 59% to 69% of children), although without statistical significance.

Allergic disorders display a clinical evolution during childhood and adolescence, related from an immunological viewpoint to the appearance of sensitizations to food allergens during the first years of life, followed by the onset of sensitization to inhalant allergens. The number of allergen sensitizations increase from childhood, when mono- or oligo-sensitizations are common, to adolescence, when polysensitization is more frequent [35,36]. This concept may be at the basis of the different frequency of sensitization to rPhl p1 and rPhl p 5 in children with different ages. According to the majority of studies, rPhl p 1 causes IgE reactivity in more than 90% of allergic subjects and exists in all grass species [22], while the rate of detection of rPhl p 5 is 65%**-**85% among populations of individuals allergic to grass pollen from temperate regions [31]. The predominant role of rPhl p 1 that dominates the immune response to grass pollen extract may explain the detection of Phl p 1 IgE at any level of grass pollen IgE and at all ages, detectable also in very young children, in whom IgE against rPhl p 5 have poor relevance.

rPhl p 7 sensitization is insignificant in our population and is completely absent in patients aged < 5 years.

The frequency of sensitization to rPhl p 12 increased with the rise of the age (from 30% to 36% ones), although without statistical significance.

Scala *et al.,* using allergen-based microarray for the detection of IgE-related sensitization to panels of allergens, gave a more precise and comprehensive evaluation for an IgE-based epidemiology. They observed that seven thousand two hundred and forty-six patients (44.16% of the total allergic cohort; 51% female) were IgE reactive on ISAC to at least one of the timothy grass pollen allergens as representative markers of other homologous grass allergens. Phl p 1 was the allergen most frequently recognized. The mean age was 30.1 ± 16.5 years, significantly higher if compared with mite allergen IgE-reactive subjects (p < 0.001), with the highest value of IgE recognition reached between 15 and 25 years of age. Phl p 2, 5, and 6 showed a similar behaviour of IgE recognition, whereas the pan-allergens Phl p 7 and Phl p 12 showed a different pattern of IgE recognition, because they did not generate any cluster with the molecules belonging to their parental biological source. Pan-allergens showed clustering trends with their homologous molecules suggesting different pathways of primary immunological sensitization [37].

Profilins (Phl p 12, Bet v 2) occur as highly Cross-Reactive allergens in a variety of plants unrelated from a botanical point of view and plant products. Patients producing specific IgE antibodies are either sensitized or at risk of developing allergic reactions to various plant pollens and plant-derived foods [38].

In our pediatric population, children with IgE against “Species Specific” and Cross-Reactive allergens were more frequently sensitized to aeroallergens Parietaria judaica and Olea and to foods peanuts and tomato compared to patients with IgE against “Species Specific” allergenic molecules only, with statistical significance. It is possible that this difference can be related to a sensitization for Cross-Reactive molecules. Indeed, among patients sensitized to “Species Specific” and Cross-Reactive molecules, only 50% were really allergic to Parietaria (IgE against Parietaria + IgE against Par j 2), while among patients only sensitized for “Species Specific” Pp allergens, 88% were really sensitized to Parietaria. It is also very remarkable that patients with IgE only against “Species Specific” allergenic molecules of Pp had not IgE vs Bet v 2, another profilin.

Considering possible correlations between clinical symptoms and the pattern of sensitization to allergenic molecules of Pp, we have observed that patients also positive for Cross-Reactive allergens were more often affected with anaphylaxis, urticaria and angioedema caused by peanuts, tomato and fruits. Further studies are needed to clarify the possible role of Cross-Reactive allergens in the development of anaphylactic symptoms due to plant-related foods.

The international literature largely reports that only a limited number of recombinant timothy grass pollen allergens account for a high percentage of IgE against grass pollen extract; it is suggested the possible utility of recombinant allergens not only for *in vitro* diagnosis, but probably also for specific immunotherapy [39]. With the use of defined molecules instead of crude allergen extract–based mixtures, it would be possible to know more precisely the mechanisms underlying immunotherapy and to develop new forms and perhaps prophylactic immunotherapy strategies [3].

In agreement with this concept, a very recent study by Tripodi et al. defined the compatibility of the profiles of IgE sensitization to Pp with a mixture of recombinant allergenic molecules of Pp previously proposed for specific immunotherapy. Sera reacting against Pp were tested for rPhl p 1, rPhl p 2, rPhl p 4, nPhl p 4, rPhl p 5b, rPhl p 6, rPhl p 7, rPhl p 11, and Phl p 12 and the IgE individual sensitization profiles were matched against an experimental allergen-specific immunotherapy preparation containing Phl p 1, Phl p 2, Phl p 5, and Phl p 6. The authors concluded that molecularly designed immunotherapy preparations tailored to patients’ needs should consider the high heterogeneity of IgE sensitization profiles to Pp, suggesting that trials are needed to test whether different molecular sensitization profiles to grass pollen underlie different clinical responses to the same immunotherapy preparation [40].

Our data show the predominant role of rPhl p 1 in pediatric populations as the most relevant sensitizing allergen detectable at all ages and at all levels of timothy grass pollen-specific IgE antibodies, while the frequency of rPhl p 5 rises with the increase in patients’ age and with grass pollen IgE levels, supporting the possible use of both allergens to create an immunotherapy tailored to the single patient.

## Conclusions

In conclusion, the assessment of sensitization to grass pollen allergenic molecules could become an important tool to achieve a better characterization of allergic sensitization in grass pollen allergy in children, which may be different in every patient, and also to give a more specific and effective immunotherapy based on sensitization to allergenic molecules.

## Abbreviations

Pp: Phleum pratense; RAST: Radioallergosorbent test; SS: Species Specific; SSCR: Species Specific and Cross Reactive.

## Competing interests

The authors have no financial involvement or relationship as employment, consultancies, honoraria, stock ownership or options, expert testimony, grants or patents received or pending, royalties, with any organization or entity with a financial interest in or financial conflict with the subject matter or materials discussed in the manuscript. The manuscript was carefully proofread and corrected for proper American spelling, grammar, and syntax by a native English speaker.
